# New evidence for a role of DANCR in cancers: a comprehensive review

**DOI:** 10.1186/s12967-024-05246-z

**Published:** 2024-06-14

**Authors:** Rong Yuan, Zhao-jun Xu, Sheng-kang Zhang, Xian-ya Cao, Ai-guo Dai, Lan Song

**Affiliations:** 1 School of Medicine, Hunan University of Chinese Medicine, 300 Xueshi Road, Hanpu Science and Teaching Park, Changsha, 410208 Hunan China; 2 Hunan Provincial Key Laboratory of Vascular Biology and Translational Medicine, 300 Xueshi Road, Hanpu Science and Teaching Park, Changsha, 410208 Hunan China; 3 Department of Cardiothoracic Surgery, the First Affiliated Hospital, Hunan University of Chinese Medicine, 97 Shaoshan Road, Changsha, 410007 Hunan China; 4 Department of Respiratory Diseases, School of Medicine, Hunan University of Chinese Medicine, Changsha, 410208 Hunan China; 5 Department of Respiratory Medicine, The First Affiliated Hospital of Hunan University of Chinese Medicine, Changsha, 410021 Hunan China; 6 Department of Biochemistry and Molecular Biology, School of Medicine, Hunan University of Chinese Medicine, Changsha, 410208 Hunan China

**Keywords:** Long noncoding RNA, DANCR, cancer, Biomarker, Resistance, Mechanism

## Abstract

Cancer remains a leading cause of mortality and poses a substantial threat to public health. Studies have revealed that Long noncoding RNA DANCR is a cytoplasmic lncRNA whose aberrant expression plays a pivotal role in various cancer types. Within tumour biology, DANCR exerts regulatory control over crucial processes such as proliferation, invasion, metastasis, angiogenesis, inflammatory responses, cellular energy metabolism reprogramming, and apoptosis. By acting as a competitive endogenous RNA for miRNAs and by interacting with proteins and mRNAs at the molecular level, DANCR contributes significantly to cancer progression. Elevated DANCR levels have also been linked to heightened resistance to anticancer drugs. Moreover, the detection of circulating DANCR holds promise as a valuable biomarker for aiding in the clinical differentiation of different cancer types. This article offers a comprehensive review and elucidation of the primary functions and molecular mechanisms through which DANCR influences tumours.

**Supplementary Information:**

The online version contains supplementary material available at 10.1186/s12967-024-05246-z.

## Introduction

The role of long noncoding RNAs (lncRNAs) in cancer has garnered increasing attention. LncRNAs are defined as noncoding RNAs exceeding 200 nucleotides [1]. Accumulating evidence indicates their involvement in regulating biological processes across various cancers [2], potentially exerting tumour-suppressive and tumour-promoting functions. The expression level of lncRNAs is related to tumorigenesis, tumour invasiveness, and the tumour stage, making them potential targets for cancer treatment. Differentiation antagonizing nonprotein coding RNA (DANCR or ANCR) is an oncogenic lncRNA that plays a crucial role in regulating tumours. DANCR was first found to be related to the dedifferentiation of epidermal cells in 2012 and is downregulated during the differentiation of progenitor cells. DANCR, an 855-base pair lncRNA, is notably downregulated during differentiation processes and is positioned on chromosome 4 in humans; it comprises three exons, with introns 1 and 2 housing a microRNA (MIR4449) and a small nucleolar RNA (SNORA26) [3]. Aberrant DANCR expression has been observed in various tumour types, prompting extensive investigations into its functional roles and molecular mechanisms. Numerous studies have indicated that the molecular mechanism of DANCR closely correlates with sustained proliferation signals [4], invasion and metastasis [5, 6], angiogenesis [7], the inflammatory phenotype [8], energy metabolism reprogramming [9], and resistance to cell death [10] in tumours, and has great value in clinical diagnosis. This review focuses on the biological function, regulatory mechanism, and clinical significance of DANCR in tumours. The aim is to provide new directions for the diagnosis and treatment of cancer in the clinic.

## Biological function of DANCR in tumours

**Fig. 1:**
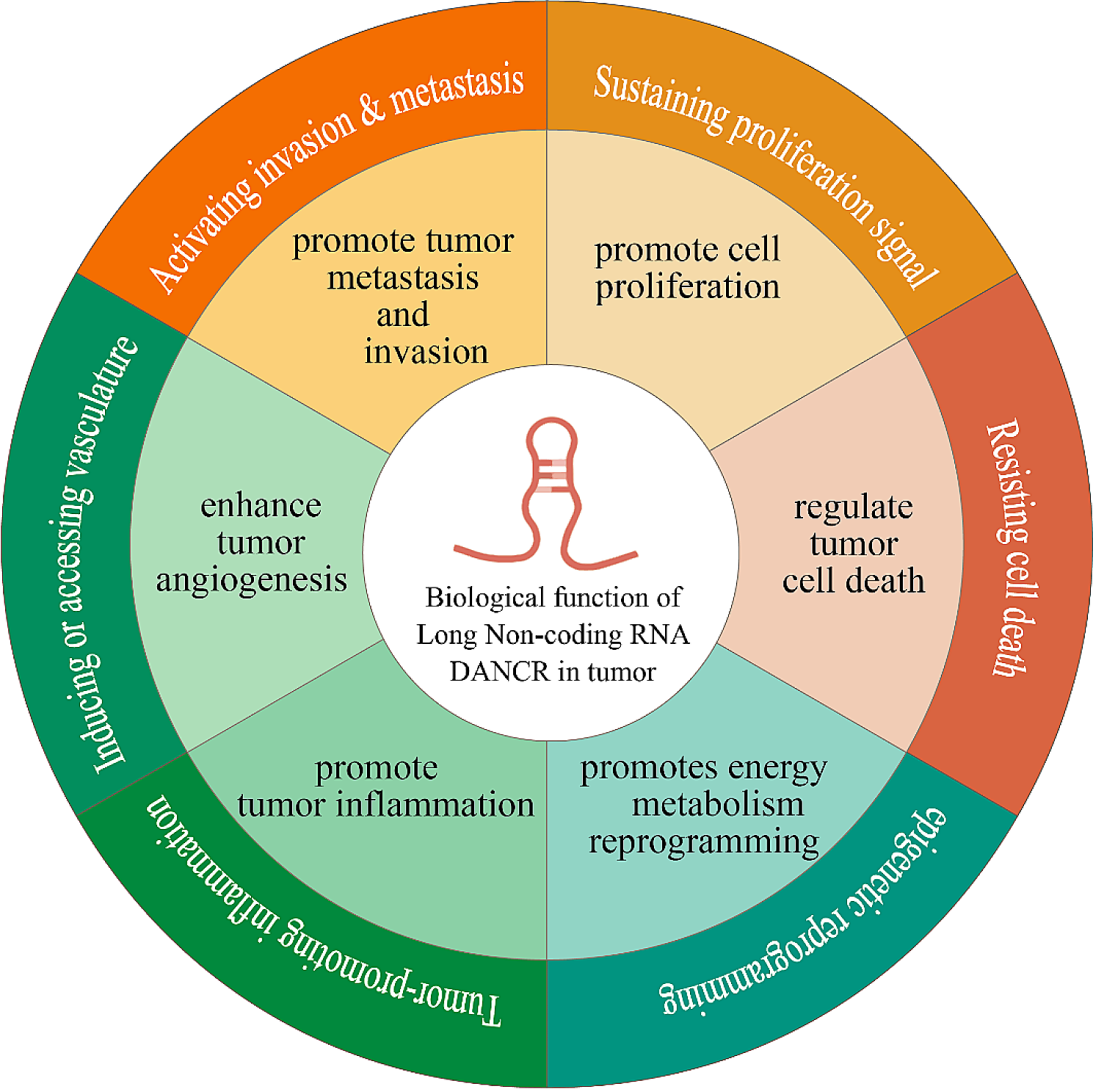
Biological function of DANCR in tumours

The biological function of DANCR in tumours encompasses various critical aspects. Among the identified tumour hallmarks, DANCR is intricately linked to abnormal proliferation, invasion, metastasis, angiogenesis, the inflammatory phenotype, energy metabolism reprogramming, and resistance of tumour cells to apoptosis (Fig. 1).

### DANCR promotes cell proliferation

Cancer cells are characterized by their sustained proliferative signalling [11]. Studies have revealed that the upregulation of DANCR in various tumours promotes proliferation by modulating the cell cycle [12–14]. Arresting the cell cycle provides additional time for repair, reducing the mutation risk and preventing tumour initiation. Research has shown that inhibiting DANCR in mouse liver cancer prolongs tumour formation cycles and reduces the tumour volume, significantly impeding tumour growth [15]. Similarly, in colorectal cancer (CRC) cells, decreased DANCR levels decrease the percentage of cells in G2 phase, inducing cell cycle arrest and impeding tumour cell proliferation [16].

### DANCR promotes tumour metastasis and invasion

Metastasis, the spread of cancer cells to distant organs, represents the deadliest phase of advanced cancer, accounting for 90% of cancer-related deaths. The invasion-metastasis cascade involves multiple discrete steps [17]. The epithelial-mesenchymal transition (EMT), a crucial process that converts epithelial cells into mesenchymal cells, promotes the invasion and dissemination of tumour cells [18]. Studies have shown that DANCR competitively binds to miR-874-3p, positively regulating SOX2 and enhancing the EMT in triple-negative breast cancer (TNBC) cells, promoting migration and invasion [5]. Moreover, evidence suggests that tumour metastasis relies on a small population of cancer stem cells with self-renewal abilities within tumours [19]. Knockdown of DANCR reduced lung cancer cell stemness and migration [20] but increased liver cancer cell stemness, facilitating intrahepatic and extrahepatic tumour colonization [19].

### DANCR enhances tumour angiogenesis

As tumours proliferate, they demand increased oxygen and nutrients, necessitating new blood vessel formation for sustained growth [21]. Recent studies have revealed a significant association between DANCR expression and tumour angiogenesis. For example, dysfunction of ZNF750 in esophageal cancer (EC) cells can trigger DANCR upregulation, leading to enhanced angiogenic properties in human umbilical vein endothelial cells (HUVECs) and human arterial endothelial cells (HAECs) [7].

### DANCR promotes tumour inflammation

The promotion of inflammation by cancer is a well-recognized hallmark [11]. Chronic inflammation activates the NF-kappa B and STAT3 signalling pathways, which are vital for tumour initiation and development. DANCR has emerged as a potent modulator of inflammation, influencing the inflammation-induced EMT and stemness in TNBC. Elevated DANCR levels notably intensify the inflammatory phenotype in breast cancer (BC) cells [8].

### DANCR promotes energy metabolism reprogramming in tumour cells

Most cancer cells rely on glycolysis, even under sufficient oxygen, following the Warburg effect for proliferation advantage [22]. Recent research has linked DANCR with cancer cell glycolysis levels. Key glycolytic enzymes, such as glucose transporter type 1 (GLUT1) and pyruvate kinase M (PKM), are influenced by DANCR, regulating glycolysis and proliferation in acute myeloid leukaemia (AML) cells through the HIF-1α/GLUT1 pathway via the miR-4701-5p/PKM axis [9].

### DANCR regulates tumour cell death

Tumour cells adeptly evade apoptosis to survive and proliferate malignantly. However, the impact of DANCR on cell death mechanisms is noteworthy. In BC cells, the inhibition of DANCR promotes apoptosis, hindering BC cell occurrence and proliferation [23]. Conversely, in prostate cancer (PC) cells, DANCR overexpression impedes apoptosis and promotes malignant proliferation through competitive miR-214-5p inhibition [10]. Moreover, autophagy can remove harmful proteins and excessive or damaged organelles in cells, preventing cytotoxicity and causing the malignant transformation of cells [24]. In glioma cells, DANCR is positively correlated with autophagy, enhancing malignant progression by upregulating ATG7 protein expression and promoting autophagy-mediated malignant transformation [25].

## The upstream and downstream regulatory mechanisms of DANCR in tumours

### Downstream regulatory mechanisms

The regulation of DANCR, a typical lncRNA, in tumours involves a multitude of intricate mechanisms. Its downstream regulatory mechanisms can be attributed to its capacity to interact with miRNAs, mRNAs, or proteins.

The competitive endogenous RNA (ceRNA) theory posits that RNA molecules containing miRNA recognition elements (MREs) can act as ceRNAs by competitively sequestering miRNAs, thereby modulating miRNA activity [26]. DANCR, which is known to bind to nearly 70 miRNAs, serves as a ceRNA for miRNAs, antagonizing their function by downregulating miRNA levels. This interaction significantly influences tumour initiation and progression. Additionally, DANCR has been shown to interact with proteins and mRNAs. For example, DANCR regulates endothelial lipase (EL/LIPG), a pivotal modulator of tumour cell metabolism, by binding to LIPG and modulating the stable expression of the LIPG protein in TNBC cells [27]. Moreover, studies have indicated that DANCR enhances the stability of the PSMD10 mRNA by interacting with the PSMD10 3’-UTR to counteract the inhibitory effects imposed by miRNAs such as miR-214 and miR-1254 [28]. Furthermore, DANCR can influence gene expression through epigenetic modifications. In cholangiocarcinoma (CCA) cells, DANCR binds to EZH2 to facilitate histone methylation of the FBP1 promoter, ultimately suppressing FBP1 expression via epigenetic mechanisms [29].

### Upstream regulatory mechanisms

**Fig. 2:**
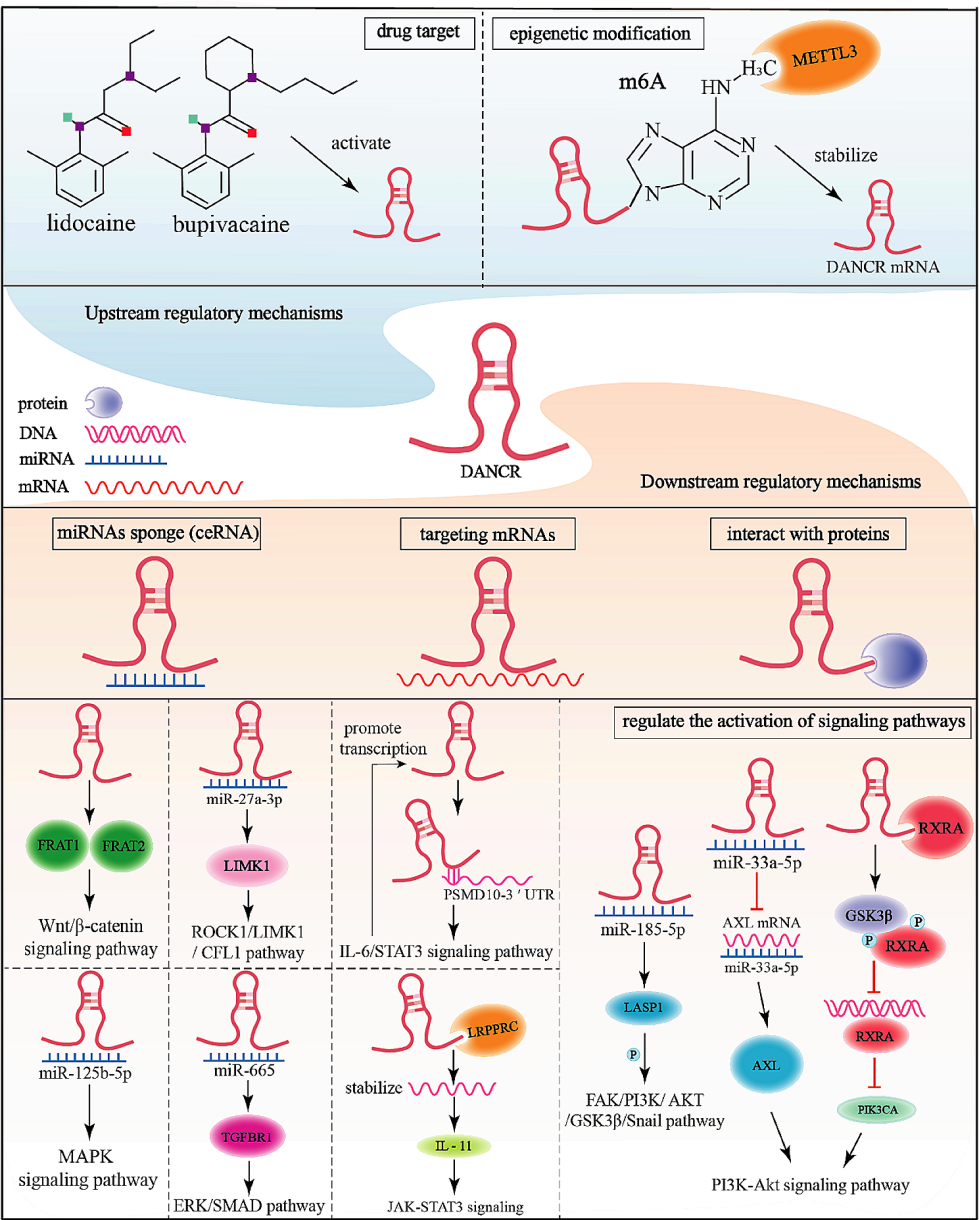
The regulatory mechanisms of DANCR in cancer. Drugs and methylation modification can regulate DANCR expression. DANCR can act as an miRNA sponges, interact with mRNAs and proteins, and ultimately activate many cancer-related signalling pathways

Moreover, the upstream regulatory mechanisms of DANCR are diverse and complex. DANCR may represent a potential drug target. Studies have confirmed that lidocaine and bupivacaine inhibit BC progression by activating the DANCR/miR-187-5p/MYB regulatory axis [30]; in addition, in terms of epigenetic modifications, Zhou et al. discovered that METTL3 enhances the stability of the DANCR mRNA through the m6A modification to promote osteosarcoma (OS) progression [31]. In summary, the regulatory mechanisms of DANCR in cancer are multifaceted and hold significant promise for cancer research and treatment (Fig. 2).

## Detailed roles of DANCR in different cancers

DNACR plays crucial roles in the development and progression of numerous tumours, as well as drug resistance (Table 1).

**Table 1:** DANCR targets and signalling pathways in a variety of cancers

Cancer type	Expression	Cell line	Regulated molecules				Function	References	
			mRNA	miRNA	protein	signal pathways			
Nasopharyngeal carcinoma	up	CNE1, CNE2, SUNE1, C666-1, HONE1, HNE1, NP-69			PTen, EZH2		∆ DANCR: ↓ cell growth and radiation resistance	[114]	
		SUNE-1, 5-8 F			PTen, EZH2		∆ DANCR: ↓ cell growth and migration	[119]	
		C666-1, SUNE-1, HNE-1, CNE1, CNE2, NP69	SOX2 mRNA		RBM3		∆ DANCR: ↓ proliferation, colony formation	[115]	
		C666-1, SUNE1, 5-8 F, NP-69		miR-4731-5p	NMT1		∆ DANCR: ↓cell migration, invasion, EMT process and cell stemness	[120]	
		SUNE-1, HONE-1, CNE-1, CNE-2, HNE-1, 5-8 F, 6-10B, C666-1, S18, S26			HIF-1α, NF90/NF45 complex		∆ DANCR: ↓ migration and invasion	[121]	
		5-8 F, SUNE-1, C666-1, NP69			PTEN, AKT		∆ DANCR: ↓ proliferation, colony formation, and migration, and ↑ apoptosis	[100]	
		neuroblastoma cells		miR-338-3p	B4GALT3		∆ DANCR: ↓ proliferation and ↑ apoptosis	[122]	
Non-small cell lung cancer	up	A549, H1299, H358, HEK 293T cells, HBE		miR-496	mTOR		∆ DANCR: ↓ proliferation, migration, invasion and ↑ apoptosis	[32]	
		BEAS-2B, NCI-H1299, A549, NCI-H1975		miR-216a	ELF4B, JAK2, MALAT1		∆ DANCR: ↓ proliferation and colony formation	[33]	
		SPC-A, NCL-H1650, NCL-H1975, SK-MES-1, A549, NCL-H358, NCI-H1299, 16HBE		miR-758-3p			∆ DANCR: ↓ viability, proliferation and ↑ cell cycle arrest	[4]	
		A549, H1299, H358, BEAS-2B			EZH2, p21		∆ DANCR: ↓ proliferation, migration, invasion EMT process, ↑ apoptosis and cell cycle arrest	[35]	
		NHBE, HEK-293T, A549, H1299, H460, SK-MES-1, Calu-3	Sox4 mRNA	miR-138			∆ DANCR: ↓ proliferation, migration, invasion EMT process, and ↑ apoptosis	[36]	
		16HBE, A549, SPCA1, H1299, H358		miR-214-5p	CIZ1		∆ DANCR: ↓ proliferation and ↑ apoptosis	[34]	
		A549, H1975, H1755, H1944, H2087, H358, H661, H1299		miR-216a		Wnt/β-catenin pathway	∆ DANCR: ↓ proliferation, stemness, migration, invasion	[20]	
		A549, SPCA1, H1299, H1975, 16HBE		miR-1225-3p	ErbB2		∆ DANCR: ↓ Migration and Invasion	[6]	
		16HBE, SPCA1, A549, H1299, H1975			HMGA2		∆ DANCR: ↓ invasion ↑↑ DANCR: ↑ invasion via increasing HMGA2	[123]	
		MM cells		miR-135b-5p	KLF9		∆ DANCR: ↓ proliferation, migration, and invasion	[124]	
	down	NCI-H23, NCI-H522			TGF-β1		↑↑ DANCR: ↓ cell migration, invasion and TGF-β1 expression	[37]	
Hepatocellular carcinoma	up	HCC cells	CTNNB1-3 ʹ UTR	miR214, miR320a, miR-199a			DANCR is involved in stemness features of HCC	[19]	
						β-catenin signalling	∆ DANCR: ↓ β-catenin pathway, proliferation and metastasis	[40]	
		Huh7, HepG2, LO2		miR-216a-5p	KLF12		∆ DANCR: ↓ proliferation, migration, invasion and ↑ apoptosis	[41]	
		MHCC-97H, Huh7, HCC‐LM3, HepG2, MHCC‐97L, Hep3B, SMMC‐7721, LO2		miR-27a‐3p	LIMK1	ROCK1/LIMK1/COFILIN1 pathway	∆ DANCR: ↓ proliferation, and metastasis	[42]	
		HEK-293T, Huh7, Hep3B	PSMD10-3 ʹ UTR	miR-214, miR-1254, miR-199a, miR-605		IL-6/STAT3 signalling	promote sorafenib resistance	[28]	
				miR-140-3p	HNRNPA1		∆ DANCR: ↓growth and metastasis	[45]	
		Bel7407, Hep3B, HepG2, Huh7, MHCC97H, LO2	ATG7	miR-222-3p			∆ DANCR: ↓ proliferation and autophagy	[43]	
		HepG2, SMMC-7721, Huh-7, Hep3B, HHCC, LO2		miR-125b-5p		MAPK signalling	∆ DANCR: ↓ migration, invasion	[44]	
		Hep G2, Hep3B, SK-Hep-1, Huh7					∆ DANCR: ↓occurrence and development of HCC	[15]	
Colorectal cancer	up	M5, HCT116, LOVO, SW620, CACO-2, DLD1, HT29, SW480, HIECs			EZH2		∆ DANCR: ↓invasion and migration	[62]	
		HT29, HCT116, SW480, LOVO, NCM460		miR-577	HSP27		∆ DANCR: ↓ proliferation and metastasis	[61]	
		LOVO, SW480, HCT116, SW620, HT29			KAT6A/ H3K9ac/p15, p16, p21		∆ DANCR: ↓ proliferation, cell cycle progression, and tumorigenesis	[16]	
		HT29, HCT116, SW116, Caco-2, FHC		miR-518a-3p	MDM2	Smad2/3 signalling pathway	∆ DANCR: ↓ proliferation, viability, metastasis	[63]	
		-		miR-145-5p	NRAS		-	[65]	
		HCT116, RKO, SW620, HT-29, LOVO			QK, MALAT1		inhibit Doxorubicin-induced apoptosis	[67]	
		HT29, SW620, HCT116, SW480, DLD-1, CRL-1790		miR-125b-5p	HK2		∆ DANCR: ↓ glycolysis rate and ↑ cisplatin sensitivity	[66]	
		NCM460, LOVO, SW620, SW480, HT29	HMGA2 mRNA	miR-185-5p	HMGA2		∆ DANCR: ↓ proliferation, migration, invasion and cell cycle progression, and ↑ apoptosis	[12]	
		HT-29 and FHC					∆ DANCR: ↓ proliferation, migration, invasion EMT process, and metastasis	[125]	
Oral squamous cell carcinoma	up	SCC9, SCC15, SCC25, CAL27, Tca8113, NHOKs		miR-216a-5p			∆ DANCR: ↓proliferation, migration and invasion, ↑apoptosis	[101]	
Tongue squamous cell carcinoma	up	SCC9, TSCCA, TCa-8113, CAL-27		miR-135a-5p	KLF8		∆ DANCR: ↓ proliferation, viability, migration and invasion	[102]	
Cholangiocarcinoma	up	HuCCT1, RBE			EZH2, FBP1		∆ DANCR: ↓ proliferation, migration	[29]	
		HuH28, HuCCT1, SG231, H69	Twist1-wt 3 ' UTR	miR-345-5p			∆ DANCR: ↓ proliferation, migration, invasion, EMT and angiogenesis and ↑ apoptosis	[103]	
Esophageal carcinoma	up	ECA109, TE-1					∆ DANCR: ↓ proliferation, migration, invasion, and ↑ apoptosis	[104]	
		EC9706, EC109, EC1, KYSE150, Het-1 A		miR-33a-5p	ZEB1		↑↑ miR-33a-5p (a target of DANCR): ↓ proliferation and metastasis	[126]	
		SHEE, KYSE140, KYSE150, KYSE180, KYSE410, KYSE510, Colo680N, ECA109		miR-4707-3p	ZNF750, FOXC2		ZNF750 induces DANCR expression, resulting in enhanced FOXC2 signalling and angiogenesis	[7]	
Gastric cancer	up	GES-1, BGC-823, MGC-803, HGC-27, MKN-45			SALL4	β-catenin signalling	∆ DANCR: ↓ proliferation, migration, invasion and EMT process, ↑ cell cycle arrest and apoptosis	[71]	
		SGC7901, BGC823	MDR1, MRP1		MDR1, MRP1		∆ DANCR: ↓ survival and increased apoptosis	[72]	
		THP-1, HGC-27			FoxO1		↑↑ DANCR: ↓IL-1β, IL-6, polarization of macrophages to M1, invasion, migration and tumour growth	[69]	
		SGC7901, MGC-803, NCI-N87, GES-1		miR-194	KLF5, AKT2		∆ DANCR: ↓ viability, ↑ autophagy, and apoptosis. KLF5 is involved in activating the transcription of DANCR	[70]	
		SGC7901, MGC803, MKN-45					∆ DANCR: ↓ proliferation, and ↑ cell cycle arrest	[127]	
Pancreatic cancer	up	PANC-1, SW1990, CAPAN-1, BxPC-3, AsPC-1, HPDE6-C7		miR-214-5p	E2F2		∆ DANCR: ↓ growth and metastasis	[105]	
		-			MLL3		DANCR downregulated MLL3 in advanced stages	[116]	
		AsPC-1, PANC-1, CFPAC-1, SW1990, BxPC-3, HPDE6-C7		miR-33b	MMP16		∆ DANCR: ↓ proliferation, migration, and invasion and EMT process	[128]	
		BxPC-3, MIA-PaCa-2, CFPAC-1, PANC-1, SW1990, HPDE6-C7		miR-135a	NLRP3		∆ DANCR: ↓ proliferation and invasion	[13]	
		BXPC-3, SW1990			IGF2BP2		DANCR is a novel target for IGF2BP2 through m6A modification	[129]	
Bladder cancer	up	5637, SW780, UM-UC-3, T24, SV-HUC-1		miR-149	MSI2		∆ DANCR: ↓ proliferation, migration, invasion and EMT process	[130]	
		UM-UC-3, T24, 293T	LRPPRC		CCND1	IL-11-STAT3 signalling pathway	promote BLCA progression	[106]	
		SW780, 5637, T24, UM-UC-3, SV-HUC-1		miR-335	VEGF-C		∆ DANCR: ↓proliferation, migration, invasion and lymphatic metastases	[117]	
Renal cell carcinoma	down	786-O, ACHNRCC					↑↑ DANCR: ↓ proliferation, migration and invasion, and ↑ apoptosis	[109]	
Glioma	up	U251, U118, LN229, U87MG, NHA		miR-634	RAB1A		∆ DANCR: ↓ proliferation and ↑ G0/G1 phase arrest	[85]	
		U87, U251, SGC7901, BGC823				Wnt/β-catenin signalling	∆ DANCR: ↓ proliferation, migration, and EMT process, and ↑ apoptosis	[83]	
		U87MG, U251MG, LN18, U138MG		miR-33a-5p, miR-33b-5p, miR-1-3p, miR-206, miR-613	AXL	PI3K/Akt/NF-kappa B signalling pathway	↑↑ DANCR: ↓ sensitivity of glioma cells to cisplatin. ∆ DANCR: ↑ sensitivity of glioma cells to cisplatin	[87]	
		HEB, U87, U251, LN229, T98G		miR-33a-5p			∆ DANCR: ↓ proliferation, migration, and EMT process, and ↑ apoptosis	[86]	
		SHG-44, U87MG, U118MG, U251MG		miR-216a	LGR5	PI3K/AKT/mTOR signalling pathway	∆ DANCR: ↓ proliferation, migration, invasion, angiogenesis, and ↑ phase arrest and apoptosis	[84]	
		LN229, U251, NHAs		miR-135a-5p	BMI1		∆ DANCR: ↓ proliferation, migration and invasion	[90]	
		U87, U251, SHG44, U118, 293T			EZH2, PTEN		∆ DANCR: ↓ invasion, migration, and proliferation, ↑ apoptosis	[89]	
		U87MG, HA	DLX6 3 ‘- UTR	miR-33b	ATG7		DANCR promote ATG7 protein: ↑ intracellular autophagy and proliferation and ↓ apoptosis	[25]	
		U251MG, LN229, LN18, T98G, HEK293T			IGF2BP2, FOXO1, PID1		IGF2BP2 increases DANCR stability and decreases DANCR methylation.	[91]	
Retinoblastoma	up	Weri-Rb1, Y79, SO-RB50, HXO-RB44, ARPE-19, hTERT-RPE1		miR-34c, miR-613	MMP-9		∆ DANCR: ↓ proliferation, migration, invasion, and EMT process	[14]	
Osteosarcoma	up	MG63, U2OS, SaOS2, HOS, 143B, FOB, fibroblast NIH3T3, 293T		miR-33a-5p	AXL	PI3K-Akt signalling pathway	∆ DANCR: ↓ proliferation, migration, invasion	[131]	
		Human OS cell lines, osteoblast			EZH2, p21, p27		∆ DANCR: ↓ proliferation, migration, invasion	[132]	
		MG-63, U2OS, MNNG/HOS, 143B, hFOB1.19		miR-335-5p, miR-1972	ROCK1		∆ DANCR: ↓ proliferation, migration, invasion and metastasis	[133]	
		MG-63, SW1353, U2OS, UMR-106, hFOB1.19				p38MAPK signalling pathway	∆ DANCR: ↓ migration and invasion	[118]	
		hFOB1.19, Saos-2		miR-149	MSI2		∆ DANCR: ↓ proliferation, migration, invasion	[134]	
		Saos-2, SJSA-1, MG63, HOS, U-2OS, hFOB 1.19			METT13		∆ DANCR: ↓ proliferation, migration and invasion. METTL3 regulate DANCR expression by m6A modification-mediated DANCR mRNA stability	[31]	
Acute myeloid leukaemia	up	HL60, U937, KG1a		miR-874-3P	ATG16L1		promote autophagy in HL60 cells via regulating ATG16L1inhibit	[99]	
		Molm-3, V411, THP-1, U937		miR-4701-5p	IGF2BP2, PKM		IGF2BP2 regulates the expression of DANCR.	[9]	
Ovarian cancer	up	A2780, PA-1, SKOV3, HO8910, HOEC		miR-145	VEGF		∆ DANCR: ↓ tube formation, angiogenesis, and invasion	[135]	
		IOSE-386, SKOV-3, OVCAR3, HO8910, HEY			UPF1		↑↑ DANCR: ↑ proliferation, migration via negatively regulating UPF1 level	[107]	
		A2780, SKOV3		miR-214	TGF-β, KLF5		∆ DANCR: ↓ viability, migration and invasion, and ↑ apoptosis	[136]	
		CAOV3, KOV3, A2780, IOSE80			SP1		∆ DANCR: ↓ viability, migration and invasion	[137]	
Cervical cancer	up	Endl/E6E7, H8	TGFBR1	miR-665		ERK/SMAD pathway	↑↑ miR-665 (a target of DANCR): ↓ proliferation, migration, and invasion	[112]	
		Caski, SW756, SiHa, C33A, HeLa, ME-180, End1/E6E7		miR-335-5p	ROCK1		∆ DANCR: ↓ proliferation, migration, and invasion	[138]	
		HCerEpiC, HeLa, SiHa, C-33A, ME-180			FRAT1, FRAT2	Wnt/β-catenin signalling pathway	∆ DANCR: ↓ proliferation	[139]	
		HeLa, SiHa, H8		miR-145-3p	KLF5, ZEB1		∆ DANCR: ↓ malignant behaviour	[140]	
	down	c33a, SiHa			HiF-1α		under hypoxic conditions: ↑↑ DANCR: ↓ proliferation, and HiF-1α	[113]	
Breast cancer	up	Hs578Bst, MCF-7, T47D, MDA-MB-468, MDA-MB-231			EZH2, CD44, ABCG2		∆ DANCR: ↓ proliferation and invasion	[49]	
		BT549, MCF7, T47D, MDA-MB-231, MDA-MB-453, MDA-MB-468, MCF10A			RXRA	PI3K/AKT signalling	∆ DANCR: ↓ proliferation and tumour growth	[47]	
		MCF-7, MDA-MB-231, MCF-10A		miR-216a-5p			∆ DANCR: ↓ proliferation, migration, and invasion	[48]	
		HCC1937, 1590, ZR-75-30, MDA-MB-468, MCF-10A		miR-758-3p	PAX6		∆ DANCR: ↓ proliferation and ↑ apoptosis	[51]	
		MCF10A, MCF-7, T47D, MDA-MB‐231, MDA-MB‐468			EZH2, SOCS3		∆ DANCR: ↓ viability, migration and invasion	[8]	
		MCF10A, ZR751, MCF7, SKBR3, BT474, MDA-MB-231, MDA-MB-468		miR-874-3p	SOX2		∆ DANCR: ↓ proliferation	[5]	
		MCF-7, HEK-93T		miR-187-5p	MYB		anti-tumour mechanisms of lidocaine and bupivacaine is mediated through the DANCR-miR-187-5p-MYB axis	[30]	
		MCF-7, CC9		miR-33a-5p	CCND1		-	[54]	
		-			FOXC1		FOXC1/lnc-FOXCUT/lnc-DANCR axis may contribute to the aggressive features	[141]	
		MDA-MB-468, MDA-MB-231, MCF-7			LIPG		DANCR binds to LIPG, enabling tumour cells to maintain LIPG protein stability and OXPHOS	[27]	
		MCF-10A, MCF-7, HCC38		miR-4319	VAPB		∆ DANCR: ↓ proliferation, migration, invasion, and ↑ apoptosis	[23]	
		BT549, MDA-MB-231			KLF5		DANCR promote cisplatin chemoresistance	[57]	
		HMECs, MCF7, ZR-75-1, MDA-MB-231, Hs578T, BT549			PRC2	Wnt/EMT signalling	RGD-PEG-ECO/siDANCR nanoparticles: ↓ proliferation, invasion and migration	[142]	
	down	MCF10A, MCF-7, MDA-MB-231, HEK293T			EZH2/CDK1		∆ DANCR: ↑ EMT program, migration and invasion	[53]	
		BT474, MCF7, T47D, MCF10A, MDA-MB-436, MDA-MB-231, MDA-MB-231HM, HEK293T		miR-331			ectopic expression of DANCR attenuates the TGF-β1induced EMT	[56]	
		MDA-MB-231, HM			TGF-β, RUNX2		∆ DANCR: ↑ proliferation, invasion and, tumour formation	[55]	
Prostate cancer	up	CWR22Rv1, PC-3, C4-2B			TIMP2/3, EZH2		∆ DANCR: ↓ migration and invasion. ↑↑ DANCR: ↑ invasion and metastasis	[75]	
		P493-6, PC3, DU145			MYC, p21		∆ DANCR: ↓ proliferation	[77]	
		DU145, PC3		miR-34a-5p	JAG1		∆ DANCR: ↑ sensitivity to docetaxel	[80]	
		PC3, C4-2, DU145		miR-135a			∆ DANCR: ↑ Paclitaxel Sensitivity	[78]	
		DU145, 22Rv1, RC-92a, PC-3M, RWPE-1		miR-214-5p	TGF-β		↑↑ DANCR: ↑ proliferation and migration, and ↓ apoptosis	[10]	
		C4-2, PC3, DU145, LNCaP, 22RV1, RWPE-1		miR-185-5p	LASP1	FAK/PI3K/AKT/GSK3β/Snail pathway	∆ DANCR: ↓ proliferation, migration, invasion, G1-S transition and EMT process	[76]	
		HPrEC, RWPE-1, PC3, DU145, LN96, OPCT-1		miR-33b-5p	LDHA		∆ DANCR: ↑ Taxol sensitivity	[79]	
Endometrial carcinoma	up	KLE, RL95-2, ishikawa, AN3CA, and HEC-1B		miR-214			∆ DANCR: ↓ proliferation and ↑ apoptosis	[108]	

∆: knock‑down or deletion, ↑↑: overexpression, ↑: positive effect, ↓: negative effect

### Lung cancer

Lung cancer is the foremost contributor to global cancer mortality. Early detection of non-small cell lung cancer (NSCLC), the predominant form of lung cancer, is limited by a lack of clinical symptoms. Hence, a highly sensitive and specific diagnostic marker for lung cancer is urgently needed. Research indicates elevated DANCR expression in NSCLC tissues compared to adjacent normal tissues [6, 32–34]. Elevated DANCR levels stimulate the proliferation, migration, and invasion of NSCLC cells [6, 35, 36]. Increased DANCR expression is significantly correlated with a reduced overall survival time [36] and advanced clinical stage in NSCLC patients [33].

Zhen et al. illustrated that DANCR overexpression in normal cells enhances the proliferation and growth of lung epithelial cells. Additionally, DANCR overexpression counteracts the inhibitory effect of miR-216a on oncogenes (ELF4B, JAK2, and MALAT1), promoting tumorigenesis and invasion [33]. Similarly, in NSCLC, DANCR acts as a sponge for several miRNAs, including miR-496 [32], miR-214-5p [34], and miR-1225-3 [6]; Yu et al. confirmed the interaction between DANCR and miR-216a in the A549 cell line, revealing that DANCR inhibits miR-216a and leads to the activation of Wnt/β-catenin signalling [20]. Furthermore, DANCR knockdown hindered EZH2-mediated epigenetic silencing of the p21 promoter, leading to enhanced p21 expression [35]. Bai et al.‘s in vivo tumour transplantation experiments in nude mice revealed decreases in tumour weight, volume, and growth upon DANCR knockdown [36]. Intriguingly, Wang et al. observed a significant downregulation of DANCR in biopsy and circulating samples from NSCLC patients compared to normal tissues. Their findings highlighted the regulatory role of DANCR in NSCLC using the NCI-H23 and NCI-H522 cell lines, which was partially mediated by TGF-β1 downregulation [37].

DANCR has emerged as a pivotal regulator of drug resistance in NSCLC, and Nicolescu et al. reported a notable increase in DANCR expression in gefitinib-resistant NSCLC cell lines [38]. Therefore, silencing DANCR could be a promising strategy to overcome drug resistance in NSCLC.

### Hepatocellular carcinoma

Hepatocellular carcinoma (HCC) comprises approximately 90% of liver cancer cases, and its incidence is increasing globally [39]. Recent studies have highlighted the impact of aberrant DANCR expression on HCC progression. DANCR expression is upregulated in HCC compared to adjacent liver tissues [40, 41], it enhances stemness features in HCC cells [19], and it is correlated with an unfavorable patient prognosis [28, 42–44]. DANCR suppression hindered HCC cell proliferation, metastasis, and invasion both in vitro and in vivo [40], induced apoptosis, and impeded cell cycle progression [41]. Targeting DANCR expression in xenograft tumours delays tumour growth [15].

DANCR competes with miR-216a-5p [41], miR-27a-3p [42], miR-222-3p [43], miR-125b-5p [44], and miR-140-3p [45] to modulate HCC progression. Moreover, the interaction of DANCR with heterogeneous nuclear ribonucleoprotein A1 (HNRNPA1) in HepG2 and Huh7 cells enhances HNRNPA1 expression by preventing its degradation and facilitating the EMT, invasion, and migration of liver cancer cells [45]. Additionally, DANCR competes for binding to the 3’UTRs of CTNNB1 and PSMD10, hindering the inhibitory effects of miR214, miR320a, and miR-199a on CTNNB1 and of miR-214, miR-1254, and other miRNAs on PSMD10 [19, 28].

DANCR also regulates HCC drug resistance. Liu et al. utilized BALB/c nude mice to establish a xenograft model and observed sorafenib resistance in HCC Hep3B cell-derived tumours. Studies have confirmed that DANCR is involved in a feedback loop involving the IL-6/STAT3 signalling pathway, enhancing HCC cell resistance to sorafenib [28].

### Breast cancer

BC is the most prevalent cancer globally and the primary cause of cancer-related deaths among women. TNBC, in particular, is characterized by high heterogeneity, high invasiveness and a poor prognosis [46], underscoring the critical need to identify molecular targets for BC treatment. In recent years, DANCR has emerged as playing a key role in the expression and drug resistance of TNBC. Research indicates that DANCR expression is elevated in TNBC tissues compared to adjacent normal tissues [47, 48], and its high levels are significantly associated with the histological grade, lymph node metastasis, advanced TNM stage, and reduced overall survival. Suppression of DANCR not only impedes the growth and invasion of BC cells [49] but also influences the EMT, stemness traits, and inflammatory responses [8, 50] while promoting apoptosis and autophagy [51].

The molecular mechanisms by which DANCR regulates BC are varied and complex. Research indicates that DANCR exerts its inhibitory effects by competitively binding to various miRNAs, including miR-874-3p [5] and miR-4319 [23], thereby fostering the malignant progression of BC. EZH2, a protein modulated by DANCR, suppresses gene expression by catalyzing histone H3 lysine 27 trimethylation (H3K27 methylation) at the target gene promoter [52]. The interplay between DANCR and EZH2 plays a pivotal role in regulating cancer progression. In TNBC, DANCR enhances EZH2 binding to the CD44 and ABCG2 gene promoters [49]. It also facilitates EZH2 binding to the SOCS3 promoter, leading to epigenetic SOCS3 downregulation [8], while forming a complex with EZH2 that promotes increased EZH2 binding to CDK1. This process triggers ubiquitin-proteasome degradation and EZH2 degradation, ultimately halting BC progression [53]. Moreover, DANCR interacts with RXRA, promoting its phosphorylation and activating the PI3K/AKT signalling pathway, promoting TNBC progression [47]. Yang et al. [27] highlighted the role of DANCR in preserving LIPG protein stability and mitochondrial metabolism through oxidative phosphorylation (OXPHOS) in TNBC cells by binding to LIPG endothelial lipase. Their work indicated that cynaroside, a novel LIPG inhibitor, effectively suppresses DANCR expression in TNBC cells, significantly impeding tumour formation. Additionally, Wu et al. observed a notable increase in the tumour volume and weight following DANCR knockdown in a xenograft model utilizing MDA-MB-231 cells in 6-week-old female nude mice [5]. In addition, miR-29b-1/a [54], lidocaine and bupivacaine [30] were shown to downregulate DANCR expression, inhibiting cancer cell progression.

Interestingly, Li et al. documented that elevated DANCR levels in MDA-MB-231 and MCF10A cells hinder tumorigenesis and distant metastasis in breast cancer. Conversely, DANCR inhibition promotes the EMT in human breast cancer cells. Through xenograft experiments on BALB/c nude mice,  researchers have shown that DANCR can restrain tumour growth and metastasis in vivo [53]. Notably, DANCR expression was downregulated in breast cancer tissues compared to that in matched normal adjacent tissues. Their study suggested that DANCR plays a role in preventing the TGF-β1-induced EMT. Reduced DANCR levels correlate with increased RUNX2 expression, fostering the invasion and metastasis of breast cancer cells [55]. Similarly, Jiang et al. reported that DANCR silencing significantly increases the tumorigenicity, proliferation, and invasion of breast cancer cells. Interestingly, DANCR expression was notably diminished in highly metastatic breast cancer cell lines [56].

In TNBC cells, DANCR interacts with Krüppel-like factor 5 (KLF5), inducing acetylation at the K369 site on KLF5. This process increases KLF5 protein levels, thereby conferring resistance to cisplatin (DDP) by inhibiting p27 transcription [57].

### Colorectal cancer

CRC is the third most common cancer globally and the second leading cause of cancer-related deaths, with a growing trend in mortality rates worldwide [58, 59]. In a study by Luan et al., hypoxia-immune-related lncRNAs were screened from TCGA and GEO public databases of CRC to predict CRC survival and immunotherapy responsiveness. DANCR is highly expressed in CRC specimens and plays a pivotal role in the initiation and progression of CRC through diverse mechanisms [60]. Functioning as a tumour promoter in CRC cells, DANCR expression is significantly increased in CRC tissues compared to adjacent nontumour tissues [12]. Increased DANCR levels enhance the growth and liver metastasis of CRC tumours [61]. Moreover, DANCR is significantly correlated with the TNM stage [62], histological grade and lymph node metastasis of CRC tissues. Increased DANCR expression indicates a poor prognosis [63] and aggressive advancement [64] of CRC; conversely, DANCR knockdown can impede the proliferation of CRC cells [16] and reduce the tumorigenicity, proliferation, migration and survival of CRC cells [63]. Research has shown that DANCR can serve as an independent indicator of both overall survival and disease-free survival in patients with CRC [64].

A recent study [62] indicated that suppressing DANCR could decrease EZH2 expression, leading to the inhibition of the invasion and migration of CRC cells. Furthermore, Lian et al. revealed that DANCR could enhance the acetyltransferase activity of lysine acetyltransferase 6 A (KAT6A) by binding to it, resulting in elevated KAT6A-mediated gene expression and promoting cell cycle progression in CRC. Using female BALB/c nude mice, they established a subcutaneous CRC mouse model and showed that DANCR knockout significantly impeded CRC tumour growth [16]. Both DANCR and heat shock protein 27 (HSP27) have been found to share binding sites with miR-577 targets in HT29 and LOVO cells. DANCR promotes HSP27 expression and facilitates the proliferation and metastasis of CRC cells by inhibiting miR-577 [61]. Various microRNAs, including miR-518a-3p [63], miR-145-5p [65] and miR-185-5p [12], were reported to interact competitively with DANCR, contributing to the aggressive progression of CRC.

In terms of CRC drug resistance mechanisms, studies have highlighted that DANCR notably enhances glycolysis in DDP-resistant colon cancer cells. In contrast, DANCR knockdown in colon cancer cells can directly interact with and increase the expression of miR-125b-5p, which targets hexokinase 2 (HK2), thus suppressing glycolysis and enhancing DDP sensitivity in tumour cells. The DANCR/miR-125b-5p/HK2 glycolysis axis has emerged as a promising therapeutic target to combat DDP resistance in colon cancer [66]. Additionally, Xiong et al. identified DANCR as a target suppressed by doxorubicin, leading to the inhibition of doxorubicin-induced apoptosis in CRC cells. This regulatory function involves the interplay between the DANCR and QK proteins, influencing the expression of the oncogenic long noncoding RNA MALAT1. Consequently, this study identified DANCR as a novel effector molecule in the downstream pathway of doxorubicin, addressing doxorubicin resistance across different cancer types [67].

### Gastric cancer

Gastric cancer (GC) is a prevalent malignant tumor worldwide and is the fourth most common cause of cancer-related mortality. The disease commonly manifests at advanced stages in the majority of patients, resulting in recurrent episodes that frequently lead to mortality [68]. Therefore, studies exploring new biomarkers for early GC detection and innovative treatment targets are urgently needed. Research has shown that DANCR expression in GC tissues surpasses that in adjacent tissues, with increased DANCR levels promoting tumour growth [69] and correlating with reduced patient survival rates [70]. Notably, significant associations have been observed between DANCR expression levels and the tumour size, TNM stage, lymph node metastasis, and invasion depth in patients with GC. Conversely, DANCR knockdown induces cell cycle arrest and hinders the EMT and apoptosis, thereby curtailing the proliferation, migration, and invasion of GC cells and ultimately suppressing GC growth in vivo [71].

DANCR has emerged as a pivotal factor in driving GC progression. Pan et al. reported that SALL4 activation induces DANCR expression in MGC-803 GC cells, thereby contributing to cancer promotion through the activation of the β-catenin pathway. Utilizing a male BALB/c nude mouse allogeneic transplantation model, they revealed that depleting DANCR inhibited the weight, volume, size, and proliferation of tumours derived from GC MGC-803 cells in vivo [71]. Moreover, FoxO1 has been found to stimulate macrophages to generate inflammatory factors and polarize them towards the M1 phenotype, exerting an antitumour effect. Xie et al. identified FoxO1 as a downstream target regulated by DANCR; elevated DANCR expression resulted in decreased FoxO1 expression, impeding macrophage polarization to the M1 type and ultimately fostering the invasion and metastasis of GC cells [69]. Additionally, KLF5, a zinc finger transcription factor, plays critical regulatory roles in cellular behaviour during tumorigenesis. Cheng et al. noted that suppressing KLF5 led to DANCR inhibition in male BALB/c nude mice, ultimately impeding GC progression [70].

DDP is commonly used as a primary chemotherapy agent for different stages of GC. Remarkably, DANCR exhibited increased expression in DDP-resistant GC tissues and cell lines, enhancing the proliferation and antiapoptotic activity of resistant cells such as SGC7901/DDP and BGC823/DDP cells. Conversely, suppressing DANCR can counteract the multidrug resistance observed in these resistant GC cells. Xu et al. examined the expression of genes related to multidrug resistance and reported that elevated DANCR levels substantially increased the mRNA and protein levels of MDR1 and MRP1. This discovery suggested that DANCR may play a role in the development of multidrug resistance by influencing the P-glycoprotein (P-gp) and MRP1 pathways [72].

### Prostate cancer

PC ranks among the three most prevalent cancers in men [73]. Current strategies aimed at reducing PC mortality risk overdiagnosis; hence, the quest for novel biomarkers persists to enhance PC diagnosis and management [74]. Multiple studies have confirmed the oncogenic role of DANCR in PC. In PC tissues and cells, DANCR expression is elevated compared to that in normal prostate tissues and cells. DANCR enhances the invasiveness and migratory capacity of PC cells in vitro [75]. Suppression of DANCR hampers proliferation, migration, invasion, and EMT protein expression in PC cells, consequently halting cell cycle progression [76].

Androgen deprivation therapy is the cornerstone treatment for advanced PC by impeding androgen receptor (AR) signalling through either androgen deprivation or AR antagonists. Jia et al. reported that DANCR knockdown reduces the enzalutamide-induced invasion and migration of PC cells and that targeting DANCR may be promising for preventing prostate cancer metastasis and mitigating potential side effects of AR inhibitors. These findings suggest the potential of DANCR knockout to prevent the adverse effects of AR inhibitors on PC treatment. In addition, in a nude mouse xenograft model, DANCR knockdown decreased the number of metastatic lesions formed by CW22Rv1 cells; within C4-2B and CW22RV1 PC cells, DANCR and EZH2 collaborate to hinder TIMP2/3 expression by epigenetically silencing their promoters, thereby promoting tumour metastasis [75]. Lu et al. revealed that the MYC oncogene can stimulate DANCR transcription, with DANCR subsequently limiting the expression of the cell cycle inhibitor p21. They confirmed DANCR as a direct target of MYC, inhibiting p21 expression to bolster PC cell proliferation [77]. In addition, miRNAs such as miR-214-5p [10], miR-185-5p [76], and miR-135a [78] have been shown to regulate the progression of PC by competitively binding to DANCR in PC cells.

Taxol is a commonly used anticancer drug. However, the efficacy of Taxol-based chemotherapy is limited due to the development of drug resistance. Zhao et al. reported that the expression of DANCR was increased in PC tissues and cells and that DANCR knockdown led to increased sensitivity of PC cells to paclitaxel [78]. Wang and Chen reported that DANCR expression was significantly upregulated in PC tissues and cells and in a paclitaxel-resistant PC cell line. They found that DANCR positively regulates Ldha by competitively binding to miR-33B-5P, thereby promoting paclitaxel resistance in PC cells [79]; additionally, treatments incorporating docetaxel (DTX) as a standard anticancer agent have produced survival advantages for patients with castration-resistant PC. Nevertheless, individuals undergoing repeated chemotherapy often exhibit resistance. Ma et al. reported the upregulation of DANCR in DTX-resistant PC tissues and cells. Suppression of DANCR increased the sensitivity of PC cells to DTX, as supported by experiments involving male BALB/c nude mice injected subcutaneously with PC3/DTX cells transfected with sh-DANCR. The outcomes revealed that DANCR knockout significantly impeded tumour growth, including tumour dimensions and mass. Their investigation confirmed that the DANCR/miR-34a-5p axis reinforces DTX resistance in PC cells by targeting JAG1 [80].

### Malignant glioma

Glioma is the most prevalent primary malignant brain tumour in adults [81]. It is characterized by rapid cell proliferation and angiogenesis. The majority of patients with WHO grade IV glioblastoma exhibit the highest malignancy level and poorest prognosis [82]. Numerous studies have indicated that DANCR may serve as an oncogenic factor crucial for glioma onset, progression, and other malignant traits. DANCR is significantly upregulated in glioma tissues and cell lines, and its elevated expression is correlated with an advanced tumour grade. Suppression of DANCR has been shown to impede glioma cell proliferation [83], migration, invasion, and angiogenesis [84]. Additionally, it can enhance apoptosis, induce cell cycle arrest [85], and is positively correlated with glioma cell proliferation and autophagy [25]. High DANCR expression is associated with increased malignancy and an unfavourable prognosis in glioma patients [86].

In glioma cells, DANCR functions as a competitive endogenous RNA for miRNAs such as miR-33a-5p, miR-33b-5p, miR-1-3p, miR-206 and miR-613 [87] to drive the malignant progression of glioma. Dysregulation of the Wnt/β-catenin signalling pathway promotes tumour stem cell renewal, proliferation, and differentiation [88]. Studies indicate that downregulating DANCR can mitigate the impact of Wnt/β-catenin activation on glioma cell proliferation and migration [83]. The PI3K/Akt/mTOR signalling pathway is implicated in tumour cell migration, invasion and angiogenesis. Wang et al. confirmed that DANCR can activate the PI3K/AKT/mTOR pathway to modulate glioma cell growth and metastasis [84]. Furthermore, DANCR epigenetically suppresses PTEN expression by binding to EZH2, thereby enhancing glioma cell invasion, migration, proliferation, and impeding apoptosis [89]. Feng et al. inoculated transfected cells into male BALB/c nude mice, and the results showed that DANCR knockdown significantly reduced tumour weight, tumour volume, and tumour growth [90].

DDP is employed as an adjuvant chemotherapy for glioma; however, the development of acquired resistance hampers its efficacy. Ma et al. silenced DANCR in female athymic BALB/c nude mice, and the apoptotic response to cisplatin was potentiated. Additionally, Ma et al. verified that DANCR enhances resistance to DDP by elevating AXL expression, which activates the PI3K/Akt/NF-kappa B pathway in glioma cells [87]. Etoposide is a well-tolerated chemotherapeutic utilized in glioblastoma treatment. Han et al. revealed that FOXO1 promotes PID1 expression, consequently enhancing DANCR stability through FOXO1 ubiquitination and contributing to glioblastoma cell resistance to etoposide [91].

### Acute myeloid leukaemia

Among adult acute leukaemias, AML has the highest incidence and shortest survival time. Regrettably, the survival rate has shown little improvement in recent decades [92]. The postoperative recurrence rate of AML is notably high [93], with its aetiology displaying significant heterogeneity [94]. Consequently, the identification of supplementary AML biomarkers holds substantial importance for screening, diagnosing, predicting the prognosis, and monitoring AML, along with forecasting individual responses to treatment [95].

Research studies have validated the significant expression of DANCR in populations enriched with leukaemia stem cells. Silencing DANCR in these cells diminishes their self-renewal capacity and proliferation. Furthermore, targeting of DANCR in vivo has been shown to extend the survival period of mice after continuous transplantation in a primary mouse model of AML [96]. This finding provides novel insights for AML treatment strategies. Among FLT3 mutations, internal tandem duplication (ITD) is the most prevalent. Wu et al. reported that high DANCR expression in FLT3-ITD + AML patients and cells was correlated with unfavourable patient outcomes. IGF2BP2 plays a role in stabilizing lncRNAs and enhancing their expression. Wu et al. revealed that IGF2BP2 regulates the glycolysis levels by upregulating DANCR expression, impacting the progression of FLT3-ITD + AML [9].

The ‘3 + 7 regimen’, which combines cytarabine (Ara-C) and anthracycline drugs, has served as the cornerstone of AML treatment for decades since the discovery of its efficacy in AML [97]. Numerous studies have detailed the use of Ara-C and daunorubicin for treating newly diagnosed adult patients with AML presenting with myelodysplastic syndrome-related changes and therapy-induced AML [98]. However, investigating alternative treatment options for patients who exhibit mutations associated with chemotherapy resistance is crucial. Studies have shown that elevated DANCR levels in human AML cells correlate with Ara-C resistance. Moreover, DANCR promotes autophagy in human AML cells treated with Ara-C, thereby increasing the expression of ATG16L1, a critical autophagy component. This study conclusively showed that DANCR contributes to Ara-C resistance in human AML cells by activating the miR-874-3P/ATG16L1 axis to enhance autophagy [99].

### Other types of cancer

In addition to the aforementioned exceptional cancers, DANCR is also implicated in the regulation of other types of cancer. It is upregulated in nasopharyngeal carcinoma (NPC) [100], oral cancer [101], tongue squamous cell carcinoma [102], CCA [103], EC [104], pancreatic cancer (PAAD) [105], bladder cancer (BLCA) [106], retinoblastoma [14], OS [31], ovarian cancer [107], and endometrial cancer [108] and downregulated in renal cell carcinoma (RCC) [109]. The regulation of DANCR expression is still controversial in papillary thyroid cancer (PTC) [110, 111] and cervical cancer (CC) [112, 113].

Resistance to radiation therapy remains a significant challenge in the management of advanced NPC. DANCR knockdown suppresses PTEN, a well-established tumour suppressor, thereby augmenting the radiosensitivity of NPC cells [114]. Additionally, DANCR can drive NPC tumorigenesis by interacting with RBM3, which contributes to NPC radioresistance [115]. Research on PAAD indicates a progressive increase in DANCR expression from early to advanced stages. Although MLL3 acts as a tumour suppressor protein, DANCR does not influence MLL3 regulation in early PAAD stages; however, DANCR downregulates MLL3 in advanced stages, promoting cancer progression [116]. Notably, DANCR expression is significantly increased in BLCA tissues with lymph node metastasis. Its role in modulating BLCA cell metastasis involves activating the IL-11-STAT3 signalling pathway through LRPPRC guidance to stabilize mRNAs and enhance CCND1 expression [106]. Furthermore, DANCR influences lymphangiogenesis by regulating VEGF-C expression, impacting tumour angiogenesis and progression [117]. Elevated p38MAPK levels impede metastatic tumour formation. Studies have revealed that silencing DANCR activates the p38MAPK pathway in OS cells, leading to decreased cell proliferation, migration, and invasion; conversely, inhibition of the p38MAPK pathway reverses these effects [118].

Limited research exists on DANCR in RCC. Current studies suggest that DANCR functions as a tumour suppressor in RCC, with its expression downregulated in RCC tissues. Elevated DANCR levels can suppress the proliferation, migration, and invasion of RCC cells while enhancing their apoptosis [109].

Studies have indicated a significant increase in DANCR expression in CC tissues and cells. High DANCR levels are positively correlated with the tumour size, FIGO stage, lymph node metastasis, and a poor prognosis. DANCR facilitates the proliferation, migration, and invasion of CC cells. However, Ta et al. observed downregulation of DANCR in the cervical tissue and serum of patients with HPV-negative CC compared to normal controls and patients with HPV-positive CC. They also noted that under hypoxic conditions, elevated DANCR suppressed HPV-negative CC cell proliferation by inhibiting HiF-1α [113]. In their research on PTC, Zhang et al. reported significantly lower DANCR expression in tumour tissues than in adjacent normal tissues. They further assessed DANCR expression in patients with various TNM stages of PTC and concluded that DANCR serves as an independent protective factor in TNM staging [110]. Conversely, Icduygu et al. reported markedly higher DANCR levels in PTC tissues than in adjacent normal tissues. Notably, DANCR expression did not increase significantly when the tumour diameter was ≤ 1 cm. Moreover, DANCR expression correlated with age and microcarcinoma, potentially influencing thyroid papillary carcinoma development [111].

## The diagnostic value of DANCR

LncRNAs exhibit stability in serum, plasma, and various body fluids and are resistant to endogenous ribonucleases. This resilience has positioned circulating lncRNAs as promising cancer biomarkers [143]. Numerous investigations have detected DANCR in serum, plasma, and exosomes, underscoring its utility in diagnosing, predicting the prognosis, and assessing treatment responses across different conditions (Table 2).

**Table 2:** The diagnostic and prognostic value of DANCR in cancer

Cancer	Marker	Sensitivity(%)	Specificity(%)	AUC	Distinction	Source	References
Lung cancer	DANCR	-	-	0.927	healthy population(HP) versus NSCLC	Tissues	[37]
		-	-	0.883		Plasma	
Hepatocellular carcinoma	AFP	65.4	77.7	0.744	HCC versus HP, CHB and cirrhosis	-	[40]
	DANCR	83.8	72.7	0.868		Plasma	
	AFP	55.8	76.5	0.650	HCC versus CHB and cirrhosis	-	
	DANCR	80.8	84.3	0.864		Plasma	
	DANCR(ANT)	54.0	64.9	0.531	HCV-HCC recurrence versus without recurrence after curative surgical resection	Tissues	[144]
	DANCR(T)	91.2	42.9	0.690			
	DANCR(T/ANT > 1.1)	88.9	49.1	0.740			
	DANCR(dd-PCR)	83.5	94.6	0.880		Circulating exosomes	
	DANCR(q-PCR)	68.3	85.7	0.831			
	AFP	97.6	9.1	0.408		-	
Colorectal cancer	DANCR	67.5	87.5	0.745	CRC versus colorectal polyps	Serum	[145]
	CEA	-	-	0.555			
	CA199	-	-	0.542			
	DANCR	67.5	82.5	0.747	CRC versus HP		
	CEA	40.0	85.0	0.623			
	CA200	32.5	80.0	0.573			
	DANCR + CEA	80.0	70.0	-			
	DANCR + CA199	77.5	65.0	-			
	DANCR + CEA + CA199	87.5	55.0	0.812			
	DANCR	87.5	72.5	0.732 ± 0.056	CRC versus adjacent normal tissue (ANT)	Tissues	[65]
	CYB561D2 + LINC00638 + DANCR	-	-	0.770	CRC tumour immune microenvironment low-risk versus high-risk	-	[146]
	CYB561D2 + LINC00639 + DANCR(3-years)	-	-	0.700		-	
	CYB561D2 + LINC00639 + DANCR(5-years)	-	-	0.702		-	
	CYB561D2 + LINC00639 + DANCR(7-years)	-	-	0.652		-	
Gastric cancer	DANCR	64.6	67.7	0.704	GC versus HP	Tissues	[71]
		72.7	79.5	0.816	GC versus ANT	Serum	
Oral squamous cell carcinoma	DANCR	80.0	73.3	0.748	OSCC versus ANT	Tissues	[147]
Papillary thyroid cancer	DANCR	85.3	66.2	0.823	PTC versus ANT	Tissues	[110]
	DANCR(GSE33630)	81.5	82.2	0.876			
	DANCR(GSE50901)	83.3	91.7	0.917			
	DANCR	72.4	70.8	0.704	PTC I/II patients versus PTC III/IV patients		
Brain tumours	DANCR	91.7	92.3	0.910	glioma versus meningioma	Tissues	[148]
		91.7	77.8	0.840	meningioma versus pituitary adenoma		
Breast cancer	DANCR	83.3	82.9	0.880	BC versus benign breast disease	Serum exosomes	[50]
	CA153	68.3	76.0	0.799			
	CEA	72.5	83.8	0.784			
	DANCR + CA153 + CEA	90.8	91.4	0.954			
	FOXC1	82.0	70.0	0.780	TNBC versus non-TNBC	Tissues	[141]
	FOXCUT	92.0	50.0	0.720			
	DANCR	45.0	90.0	0.650			
Ovarian cancer	DANCR	-	-	0.852	OC versus ANT	Tissues	[107]
Cervical squamous cell carcinoma	DANCR	-	-	0.907	HPV-negative CSCC patients versus HP	Tissues	[113]
		-	-	0.874		Serum	
		-	-	0.504	HPV-16-positive CSCC patients versus HP	Tissues	
		-	-	0.501		Serum	
		-	-	0.598	HPV-18-positive CSCC patients versus HP	Tissues	
		-	-	0.617		Serum	
Prostate cancer	DANCR	-	-	0.852	PC patients versus HP	Serum	[10]

Ma et al. [40] proposed that plasma DANCR levels could outperform AFP as a diagnostic biomarker for HCC. Notably, Table 2 shows the superior diagnostic accuracy of plasma DANCR levels over AFP levels in HCC patients. Furthermore, Wang et al. [144] highlighted the potential of circulating exosomal DANCR as a noninvasive prognostic marker for hepatitis C virus-related HCC. In CRC, Shen et al. investigated DANCR levels in both tissue samples and serum, revealing significant upregulation in both samples. They noted a decrease in serum DANCR expression among postoperative individuals compared to pretreatment and relapsed patients. Their study integrated carcinoembryonic antigen (CEA) and carbohydrate antigen 199 (CA199) as tumour markers and concluded that the concurrent detection of DANCR, CEA, and CA199 yielded the highest sensitivity and area under the curve (AUC) compared to the detection of a single biomarker. This comprehensive approach enhanced CRC diagnostic efficacy [145]. Research on GC indicated elevated DANCR expression levels in patient serum compared to healthy controls, with a notably high AUC value, indicating of its diagnostic potential [71]. Zhang et al. explored DANCR as a tumour marker for PTC utilizing receiver operating characteristic curves to differentiate between patients with early-stage (I/II) and advanced-stage (III/IV) PTC [110]. Additionally, Shi et al. reported significantly elevated serum exosomal DANCR levels in BC patients relative to those in patients with benign breast diseases and healthy individuals. The combined detection of serum exosomal DANCR, CA153, and CEA levels markedly improved the BC diagnostic accuracy, with serum exosomal DANCR levels serving as an independent BC risk factor [50].

These studies illustrate the considerable promise of measuring the circulating DANCR level as a novel noninvasive biomarker for cancer diagnosis. Nonetheless, existing limitations in its clinical implementation underscore the need for additional research on lncRNA liquid biopsy to reinforce its viability.

## Conclusions and prospects

**Fig. 3:**
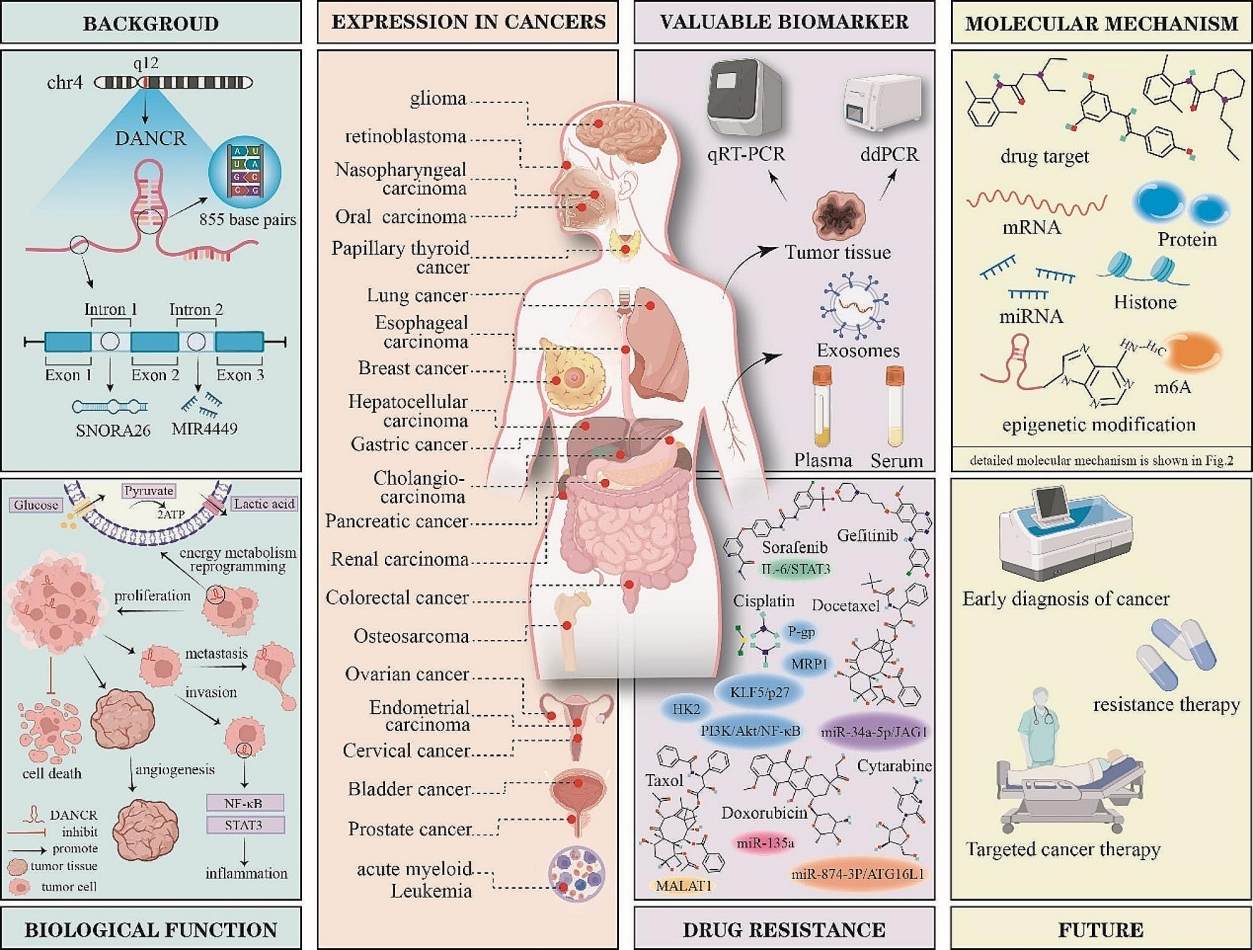
The role of DANCR in cancer. DANCR is located on chromosome 4 with 855 base pairs and consists of three exons, with introns 1 and 2 containing miR4449 and snoR26. DANCR can regulate the hallmarks of various cancers. DANCR is aberrantly expressed in a variety of tumours. Circulating DANCR has diagnostic value. DANCR regulates drug resistance in a variety of tumours. DANCR has complex molecular mechanisms. DANCR has broad application prospects

Although DANCR is generally considered an oncogenic factor in most cancers, it is considered a tumour suppressor in RCC, and its role in some cancers, such as lung cancer and PTC, remains controversial. These discrepancies suggest that the distinct expression profiles and functional roles of DANCR across various cancer types and cell lines significantly influence disease progression and clinical outcomes. Extensive research has revealed the intricate mechanisms through which DANCR promotes tumour development, involving interactions such as a ceRNA targeting microRNAs, the modulation of mRNA or protein activities, initiation of signalling cascades, and epigenetic modulation. Despite the identified role of DANCR in mediating drug resistance in multiple cancer types, investigations into its precise contribution to this phenomenon remain in nascent stages, and the underlying mechanisms remain unclear. Notably, circulating DANCR exhibits exceptional precision in discriminating among diverse cancer types. Nevertheless, conclusive clinical investigations are imperative to confirm the implications of DANCR for early-stage diagnosis, prognostication, and pharmacotherapy (Fig. 3).

Although targeting DANCR for tumour therapy holds promise, its therapeutic application encounters various obstacles, including drug-related toxicity, off-target effects, and the development of secure and effective delivery mechanisms. Concurrently, DANCR shows potential for clinical utility in addressing tumour drug resistance. A standardized research framework encompassing the isolation of circulating DANCR and the enhancement of bioinformatic sequencing techniques characterized by increased efficiency, sensitivity, and specificity must be developed to establish DANCR as a novel biomarker.

### Electronic supplementary material

Below is the link to the electronic supplementary material.

Supplementary Material 1

Supplementary Material 2

## Abbreviations

lncRNAs: Long non-coding RNAs
DANCR/ANCR: Differentiation antagonizing non-protein coding RNA
CRC: Colorectal cancer
EMT: Epithelial-mesenchymal transition
TNBC: Triple-negative breast cancer
EC: Esophageal cancer
HUVECs: Human umbilical vein endothelial cells
HAECs: Human arterial endothelial cells
BC: Breast cancer
GLUT1: Glucose transporter type 1
PKM: Pyruvate kinase M
AML: Acute myeloid leukaemia
PC: Prostate cancer
ceRNA: Competitive endogenous RNA
MREs: miRNA recognition elements
EL/LIPG: Endothelial lipase
CCA: Cholangiocarcinoma
OS: Osteosarcoma
NSCLC: Non-small cell lung cancer
HCC: Hepatocellular carcinoma
HNRNPA1: Heterogeneous nuclear ribonucleoprotein A1
OXPHOS: Oxidative phosphorylation
KLF5: Krüppel-like factor 5
DDP: Cisplatin
KAT6A: Lysine acetyltransferase 6 A
HSP27: Heat shock protein 27
HK2: Hexokinase 2
GC: Gastric cancer
P-gp: P-glycoprotein
AR: Androgen receptor
DTX: Docetaxel
ITD: Internal tandem duplication
Ara-C: Cytarabine
NPC: Nasopharyngeal carcinoma
PAAD: Pancreatic cancer
BLCA: Bladder cancer
RCC: Renal cell carcinoma
PTC: Papillary thyroid cancer
CC: Cervical cancer
CEA: Carcinoembryonic antigen
CA199: Carbohydrate antigen 199
AUC: Area under the curve
