# Supplementary material for: New evidence for a role of DANCR in cancers: a comprehensive review
Source: J Transl Med. 2024 Jun 14;22:569. doi: 10.1186/s12967-024-05246-z (PMC11177382; doi:10.1186/s12967-024-05246-z)
Supplement: Supplementary file 2 — Supplementary Material 2 [file 12967_2024_5246_MOESM2_ESM.docx]

Dear Editor，

Thank you very much for your comments and professional advice. These opinions help to improve academic rigor of our article. Based on your suggestion and request, we have made corrected modifications on the revised manuscript. Meanwhile, the manuscript had be reviewed and edited by language services of American Journal Experts. Now, I resubmit it to you and hope that the revision is acceptable. Great thanks to you and the referees for your time and effort on this paper.

With best wishes,

Lan Song

We would like to thank the reviewers for the constructive and positive comments.

Response to Reviewer #1

**comment**: In the presented manuscript the authors summarize the molecular mechanisms throught which long non-coding RNA DANCR influences tumours, offering a complete, detailed and well organized review about the functions of DANCR in several tumor types.

The presented paper focuses on a very important and interesting topic, considering the urgent need to find novel biomarkers for tumor targeting.

The whole manuscript is written in a correct English form, and the structure of the entire review is well organized, with an accurate description of the treatments currently used, giving to the reader a complete overview about the role of DANCR in tumor angiogenesis, inflammation, cell death and priliferation and other regulatory mechanisms in tumor development.

Two little minor concerns, however, could be corrected:

1. At page 16, row 39, the phrase "Jia et al. reported that DANCR knockdown diminishes the enzalutamide-induced invasion and migration of PC cells, emphasizing a potential therapeutic role in countering AR antagonist-related effects. may prevent potential side effects of AR inhibitors in treating PC" sounds as uncorrect and should be corrected.

2. At page 17, row 34, please correct the capital letter in "Additionally"

**Response**: We would like to thank the reviewer for the positive comments and careful reading.

1. As suggested by the reviewer, at page 16, row 39, we have corrected the "Jia et al. reported that DANCR knockdown diminishes the enzalutamide-induced invasion and migration of PC cells, emphasizing a potential therapeutic role in countering AR antagonist-related effects. may prevent potential side effects of AR inhibitors in treating PC" into "Jia et al. reported that DANCR knockdown reduces the enzalutamide-induced invasion and migration of PC cells and that targeting DANCR may be promising for preventing prostate cancer metastasis and mitigating potential side effects of AR inhibitors. "
2. At page 17, row 34, we changed the "Additionally" to "additionally".

Response to Reviewer #2

**Comment**: The topic is interesting and timely, however the manuscript needs a careful english revision to better appreciate the meaning of the sentences.

**Response**: Thanks for your advice. We regret for our poor work, however, we did our best to improve the manuscript, and asked the American Journal Experts for help with English revision to the manuscript. These changes will not affect the content and framework of this article, and marked in red and blue in the modified paper. We sincerely thank the reviewers for enthusiastic work and hope that the revised content will be recognized.
